# Optimized renal protection with combined thymoquinone and glycine treatment: synergistic management of streptozotocin-induced nephropathy

**DOI:** 10.1007/s00210-025-04725-4

**Published:** 2025-11-08

**Authors:** Sara S. Eldebsy, Entsar A. Saad, Rana R. El Sadda

**Affiliations:** Chemistry Department, Faculty of Science, Damietta University, Damietta, 34517 Egypt

**Keywords:** Diabetes, Thymoquinone, Glycine, Inflammation, Antioxidant, Reno-protective

## Abstract

Diabetic nephropathy is still a chief reason for morbidity and mortality in persons with renal dysfunction. Thymoquinone, a primary constituent of black seed oil extracted from *Nigella sativa*, has anti-inflammatory, antioxidant, anticancer, and antimicrobial properties. Glycine, an amino acid and neurotransmitter, participates in diverse physiological mechanisms. This study investigated the nephroprotective role of thymoquinone and glycine against streptozotocin (STZ)-induced diabetic nephropathy. Forty-two adult male Swiss albino rats were segregated into seven groups, each comprising six. These groups consisted of control normal rats; rats administered 60-mg STZ/kg (nephropathy); nephropathy rats treated with oral doses of 20-mg/kg/day thymoquinone (T20) or 30-mg/kg/day thymoquinone (T30); nephropathy rats treated with oral doses of 50-mg/kg/day glycine (G50) or 100-mg/kg/day glycine (G100); (T + G) nephropathy rats receiving combination therapy of 30-mg/kg/day thymoquinone and 100-mg/kg/day glycine. Various biochemical factors, including glutathione (GSH), total antioxidant capacity (TAC), malondialdehyde (MDA), tumor necrosis factor-alpha (TNF-α), interleukin-10 (IL-10), total and myocardial creatine kinase (CK), kidney function parameters, blood electrolytes (Na, K, Cl, Ca, and P), and kidney histopathology, were assessed. The combined therapy of thymoquinone and glycine demonstrated enhanced efficacy in improving biochemical profiles, antioxidant levels, anti-inflammatory responses, and renal structure compared to monotherapies employing thymoquinone or glycine. The confluence of thymoquinone and glycine potentially operates through manifold pathways, encompassing the regulation of oxidative stress and modulation of inflammatory cascades. This study elucidates the potential synergistic benefits of integrating thymoquinone and glycine in diabetic nephropathy management, thereby heralding novel avenues for therapeutic interventions.

## Introduction

Diabetes mellitus (DM) is a major endocrine disease and a growing health concern in nearly every country. It is one of the principal five causes of early death globally. In Egypt, DM is the most significant medical and public health challenge (Hassanien et al. 2020). The high mortality rate linked to DM is primarily caused by its numerous complications that can affect various organs. These complications include diabetic nephropathy, retinopathy, neuropathy, cardiovascular diseases, and diabetic foot ulcers (Poly et al. 2023). As a result, individuals may experience severe health issues such as kidney failure, vision loss, stroke, heart failure, and amputations (Hassanien et al. 2020; Saad et al. 2019a, 2019b). For diabetic patients, oral administration of combination therapy provides a less invasive option especially when compared to the discomfort accompanying insulin injections (Coelho et al. 2024).

Nephropathy is a serious condition that can originate from a range of underlying pathological conditions. Notably, it is frequently associated with chronic diseases such as obesity (Habib et al. 2015), cancer (Saad et al. 2017a), and DM (Saad et al. 2015). Unfortunately, using certain medications particularly chemotherapeutics (El Ezaby et al. 2024; Saad et al. 2019a, b) like cisplatin (Aboseada et al. 2021) and doxorubicin (Basal et al. 2023) or exposure to toxic substances (Saad 2013; Toson et al. 2014) can also cause nephropathy. Further, inflammation and oxidative stress contribute to developing nephropathy (Saad et al. 2017b). Understanding these connections is crucial for effective prevention and management strategies.

Specifically, diabetic nephropathy is a typical complication of type 1 and type 2 DM; from 30 to 40% of DM patients have DM-associated kidney disease, even when blood sugar levels are well managed. Diabetic nephropathy remains one of the chief causes of morbidity and death among individuals experiencing renal dysfunction. It can lead to end-stage renal disease connected with abnormalities in inflammatory responses and oxidative stress (Guo et al. 2021). Though many medications are currently used to cure it, achieving satisfactory control remains a challenge. Therefore, there is an ongoing need to develop more effective drugs. Natural products possess unique properties that may offer enhanced efficacy and safety (Abdlsamea et al. 2025), making them promising candidates for new therapies.

Thymoquinone, the principal active compound in *Nigella sativa* seeds oil, offers a variety of therapeutic benefits, having antioxidant, anti-inflammatory, anti-cancer, antibacterial, antifungal, and anticonvulsant effects (Abbas et al. 2024). Thymoquinone may protect kidneys by reducing inflammation and preventing excessive cell death (Zhang et al. 2024). Thymoquinone was shown to help improve creatinine and urea levels in nephropathy caused by rheumatoid arthritis or morphine, decrease deposits and both the size and number of renal calculi induced by ethylene glycol (Halder et al. 2024). In addition, thymoquinone could protect against diabetic complications, as highlighted in numerous research studies. It showed protective effects against diabetic retinopathy by bolstering neuroprotective factors, reducing cell death, and safeguarding the integrity of the blood-retinal barrier (Khan and Zaidi 2024). It protected superoxide dismutase (SOD) against methylglyoxal or glucose-induced glycation (Hofni et al. 2023). Additionally, it has been shown to have a neuroprotective role in diabetic peripheral neuropathy; it enhanced nerve conduction and reduced apoptosis in nerve cells (Sun and Shahrajabian 2023). It offers potential long-term benefits in managing diabetes-related neurovascular complications, insulin resistance, and glycation-induced damage (Mahdavi and Javadivala 2024). These findings suggest that thymoquinone could serve as a valuable dietary and therapeutic target.

Glycine is an amino acid with a “H” atom as a side chain group so it is considered the simplest amino acid. Our bodies can synthesize glycine, so it is a non-essential amino acid. It is primarily a neutral and metabolically inert amino acid. It is required for synthesizing many body molecules, including proteins, porphyrins, glucose, creatine, GSH, neurotransmitters, bile salts, and purine nucleotides. Additionally, as a component of GSH, glycine plays a role in antioxidant defense and detoxification reactions. Glycine also exhibits anti-inflammatory, cytoprotective, and immunomodulatory effects. Notably, it significantly reduces the release of superoxide ions and TNF-α (Shosha et al. 2023). Glycine participates in regulating the immune response by impeding pro-inflammatory cytokines production such as TNF-α, interleukin-6 (IL-6), and IL-1β, and enhancing anti-inflammatory cytokine IL-10 production in activated macrophages, leukocytes (Aguayo-Cerón et al. 2023), and T lymphocytes under pathological conditions (Raynor and Chi 2024). Glycine has cytoprotective properties that reduce inflammation by preventing immunogenic effects related to necrosis and directly inhibiting pyroptosis (Xu et al. 2024). Glycine seems important in diabetes management, as it promotes the secretion of glucagon-like peptide-1, insulin, and glucagon. Clinical investigations have demonstrated that higher levels of circulating glycine are associated with a reduced risk of developing type 2 DM (Aguayo-Cerón et al. 2023).

The primary objective of this study is to assess the combined therapeutic potential of thymoquinone and glycine in protecting the kidneys from damage induced by streptozotocin-induced diabetes in rats. According to the literature, this has not been investigated before.

## Material and methods

### Chemicals

Streptozotocin and thymoquinone were purchased from Cromonelle Lab, Cairo. Glycine (99% assay) was purchased from El Nasr Pharmaceutical Chemicals, Egypt. Other chemicals and reagents of the highest available pure grade were used.

### Experimental animals

Adult male Swiss albino rats (body weight 150–200 g, aged 6 to 8 weeks), bought from Theodor Bilharz Research Institute in Giza, Egypt, were used. The rats were acclimatized for a minimum of 7 days under standard conditions: a temperature of 23 ± 2 °C, appropriate humidity, and a 12:12 h light/dark cycle, as outlined in the “Guide for the Care and Use of Laboratory Animals” produced by the National Academy of Science and published by the National Institute of Health. The rats had ad libitum access to ordinary food and water.

### Induction of nephropathy

Experimental diabetic nephropathy was induced in rats by administering STZ (60 mg/kg) intraperitoneally in a 0.1 M cold citrate buffer, pH 4.5.

### Experimental design

Forty-two rats were divided into 7 groups (6 per group):

Normal: control normal rats.

Nephropathy: rats received one dose of STZ (60 mg/kg, i.p.).

T (20): rats received STZ (60 mg/kg, i.p.). Three days later, they were treated with thymoquinone (20 mg/kg/day, oral gavage) daily for 21 days.

T (30): rats received STZ (60 mg/kg, i.p.). Three days later, they were treated with thymoquinone (30 mg/kg/day, oral gavage) daily for 21 days (Pari and Sankaranarayanan 2009).

G (50): rats received STZ (60 mg/kg, i.p.). Three days later, they were treated with glycine (50 mg/kg/day, oral gavage) daily for 21 days.

G (100): rats received STZ (60 mg/kg, i.p.). Three days later, they were treated with glycine (100 mg/kg/day, oral gavage) daily for 21 days (Shosha et al. 2023).

T + G: rats received STZ (60 mg/kg, i.p.). Three days later, they were treated with thymoquinone (30 mg/kg/day, oral gavage) and glycine (100 mg/kg/day, oral gavage) daily for 21 days.

### Samples

Under the effect of ketamine (75 mg/kg) and xylazine (10 mg/kg) anesthesia, rats were euthanized by cervical dislocation following the final treatment, and the kidneys were collected. Blood samples were taken just before euthanasia via cardiac puncture while the rats were under anesthesia and left to clot. Sera were separated after centrifugation (15 min at 3000 rpm) of the clotted blood samples and then stored at − 20 °C. Kidney weight relative to body weight was calculated as a kidney index. A part of the separated kidneys was fixed in 10% buffered formalin for histopathology. Kidney tissue homogenates (10%, weight/volume) were prepared in ice-cold phosphate-buffered saline and centrifuged (15 min at 4000 rpm) to obtain the clear supernatant for analysis of TAC, MDA, and GSH.

### Serum parameters analysis

Biochemical parameters were measured following the manufacturer’s manual using commercially available kits: creatinine, urea, and uric acid kits were purchased from HUMAN Diagnostics, GERMANY. Creatine kinase (CK) [total (CK-T) and myocardial (CK-MB)] were purchased from SPIN REACT, Ctra, Santa, Coloma Spain. Calcium, phosphorus, chloride, sodium, and potassium concentrations were estimated by SENSA-CORE-ST-200-PLUS-BLOOD-ELECTROLYTE ANALSIS DEVICE. For inflammation analysis, serum levels of TNF-α and IL-10 were determined using ELISA immunoassay kits obtained from Wuhan Fine Biotech, China.

### Kidney homogenate parameters analysis

MDA, GSH, and TAC in kidney homogenate clear supernatant were determined based on the procedures outlined in their commercial kits purchased from BIO-DIAGNOSTIC, Giza, Egypt.

### Histopathological examination

Kidney tissue samples underwent a series of steps to prepare them for microscopic examination. First, the tissues were preserved in formalin, a hardening solution. Then, they were processed to be embedded in paraffin wax for stability. The paraffin block was sliced into thin sections, only 5 µm thick. Finally, these sections were stained with hematoxylin and eosin to differentiate cellular structures for viewing under a light microscope.

### Statistics

Statistically, results were interpreted using Statistical Package for the Social Sciences version 17 (SPSS Software, SPSS Inc., Chicago, USA) and stated as mean ± standard error (SE). For data with Gaussian distribution, one-way ANOVA followed by the Tukey test was used. Typically, a difference was deemed significant if the *p* value was less than 0.05.

## Results

**Fig. 1:**
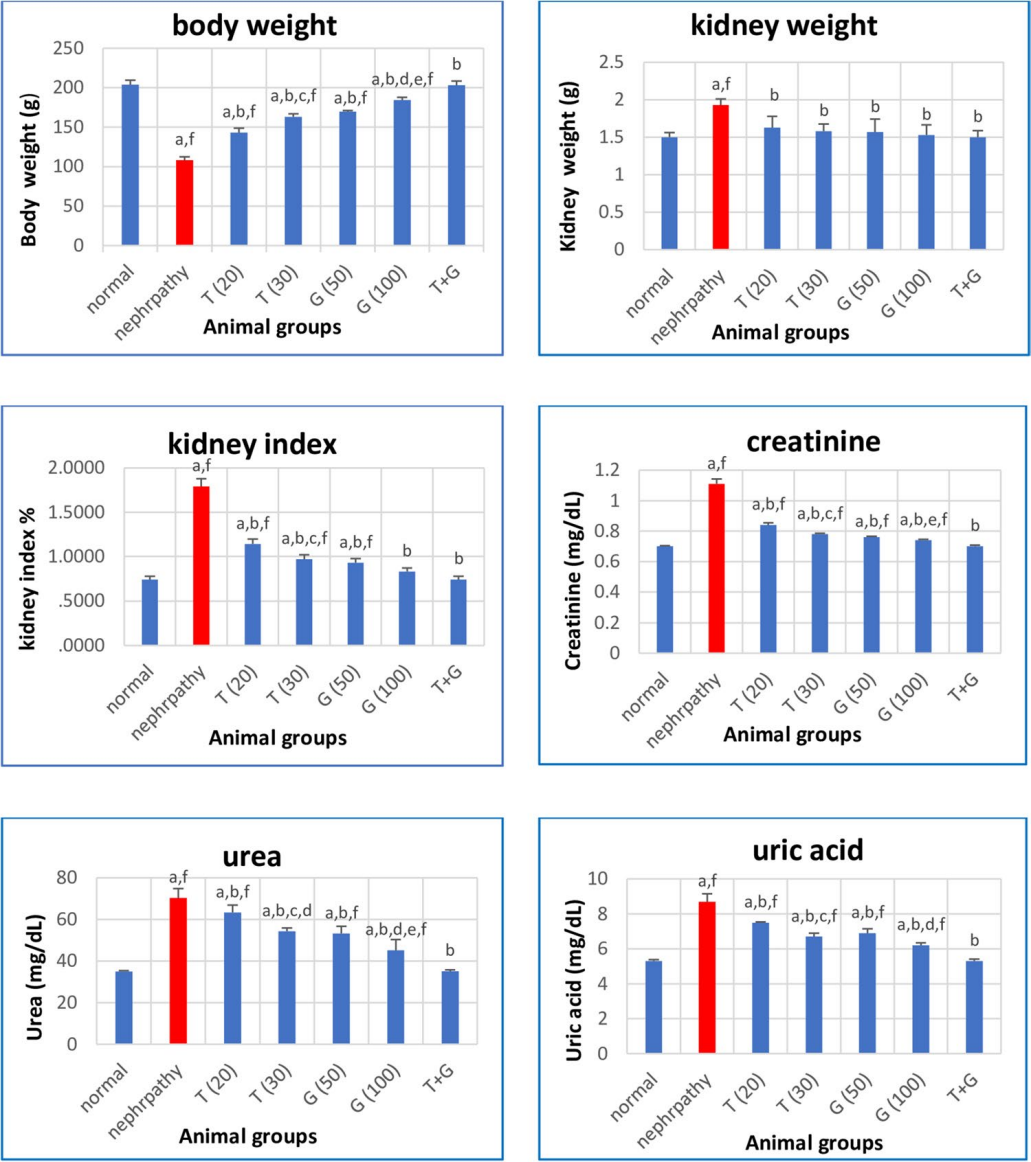
Effect of different treatments on body weight, kidney weight, kidney index, creatinine, urea, and uric acid in different study groups. Data are expressed as mean ± SE, *n* = 6 rats. Test: ANOVA test followed by Tukey test. *P* < 0.05 is significant. (a) *P* < 0.05 against normal, (b) *P* < 0.05 against nephropathy, (c) *P* < 0.05 against T (20), (d) *P* < 0.05 against G (50), (e) *P* < 0.05 against T (30), (f) *P* < 0.05 against T + G

Figure 1 illustrates that rats with nephropathy induced by STZ experienced a decrease in body weight, along with increases in kidney weight and the kidney index, compared to the normal rats (*P* < 0.05). However, treated nephropathic rats with thymoquinone alone, glycine alone, or the combination of both showed increases in body weight, accompanied by decreases in kidney weight and the kidney index (*P* < 0.05 for all treated groups compared to the nephropathy group). Statistically, significant differences (*P* < 0.05) were found between T (20) and T (30), as well as between T + G and the groups T (20), T (30), and G (50) in terms of body weight and kidney index. Additionally, significant differences (*P* < 0.05) were noted between G (50) and G (100), T (30) and G (100), and T + G and G (100) regarding body weight only.

Rats in the nephropathy group exhibited elevations in kidney function–related markers, including creatinine, urea, and uric acid (*P* < 0.05 compared to normal group levels). All these markers revealed a significant restoration towards normal values in groups treated with glycine or/and thymoquinone (*P* < 0.05 for all treated groups compared to the nephropathy group). Both thymoquinone and glycine significantly restored these markers’ levels in a dose-dependent manner, with the combined therapy showing a reno-protective pronounced effect. By comparison between different treated groups, there were significant (*P* < 0.05) differences between T (20) and T (30) in creatinine, urea, uric acid, between G (50) and G (100) in urea and uric acid, between T(30) and G (100) in creatinine and urea, and, finally, between T + G and G (100) in creatinine, urea, and uric acid (Fig. 1).

**Fig. 2:**
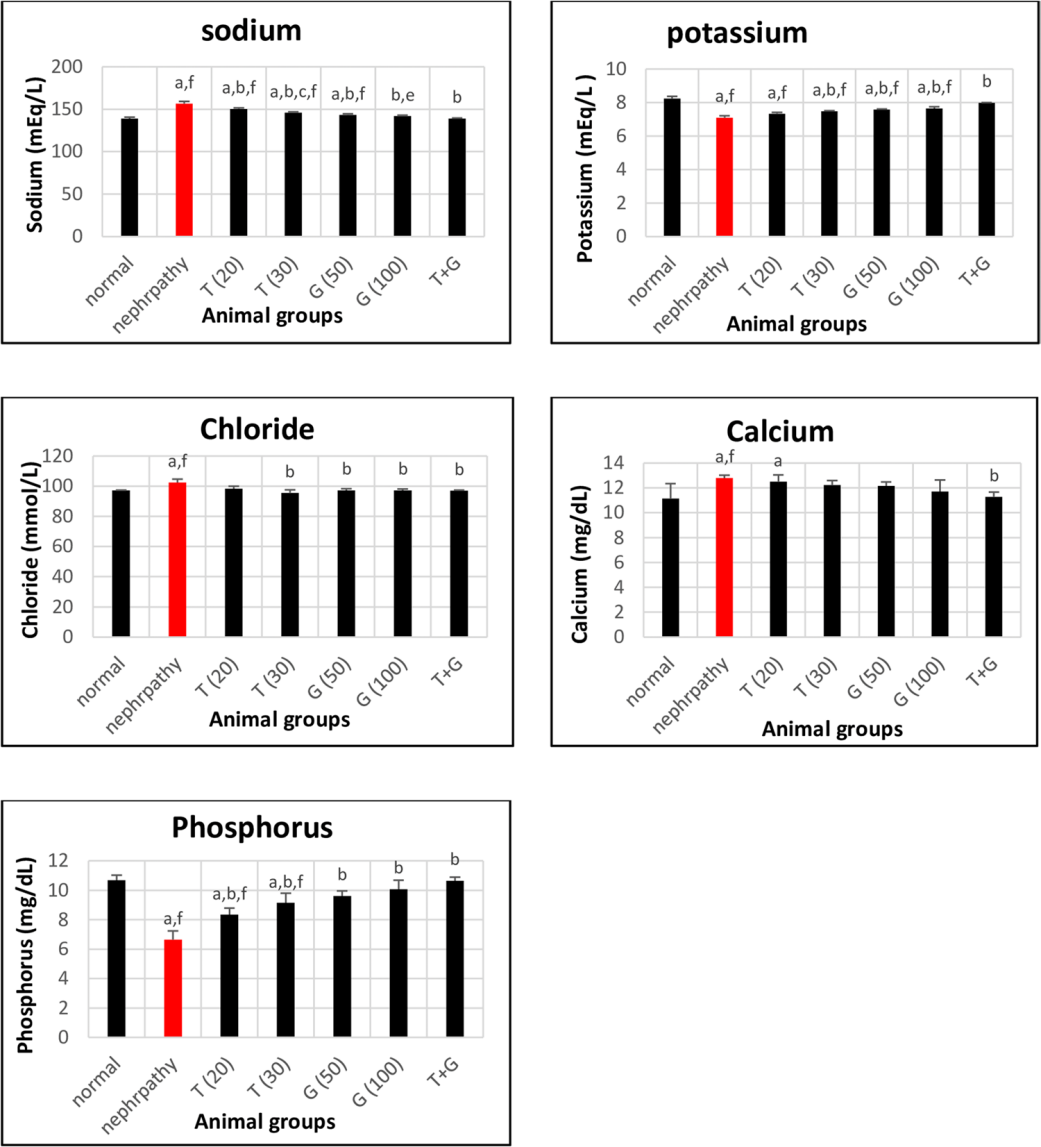
Effect of different treatments on sodium, potassium, chloride, calcium, and phosphorus levels in different study groups. Data are expressed as mean ± SE, *n* = 6 rats. Test: ANOVA test followed by Tukey test. *P* < 0.05 is significant. (a) *P* < 0.05 against normal, (b) *P* < 0.05 against nephropathy, (c) *P* < 0.05 against T (20), (d) *P* < 0.05 against G (50), (e) *P* < 0.05 against T (30), (f) *P* < 0.05 against T + G

In Fig. 2, serum minerals of STZ-induced nephropathy rats displayed significant increases in sodium, calcium, and chloride levels and decreases in potassium and phosphorus levels compared to normal control rats (*P* < 0.05). The effect of thymoquinone or/and glycine therapy on ameliorating sodium, potassium, calcium, chloride, and phosphorus levels was significant (*P* < 0.05 vs. the nephropathy group), except for T (20) effect on K, Cl, and Ca levels, as well as T (30), G (50), and G (100) effects on Ca level were insignificant (*P* > 0.05). Comparing treated groups with each other, only significant (*P* < 0.05) differences were found between T (20) and T (30) and between T (30) and G (100) in sodium levels, as well as between T + G and G (100) in potassium levels.

**Fig. 3:**
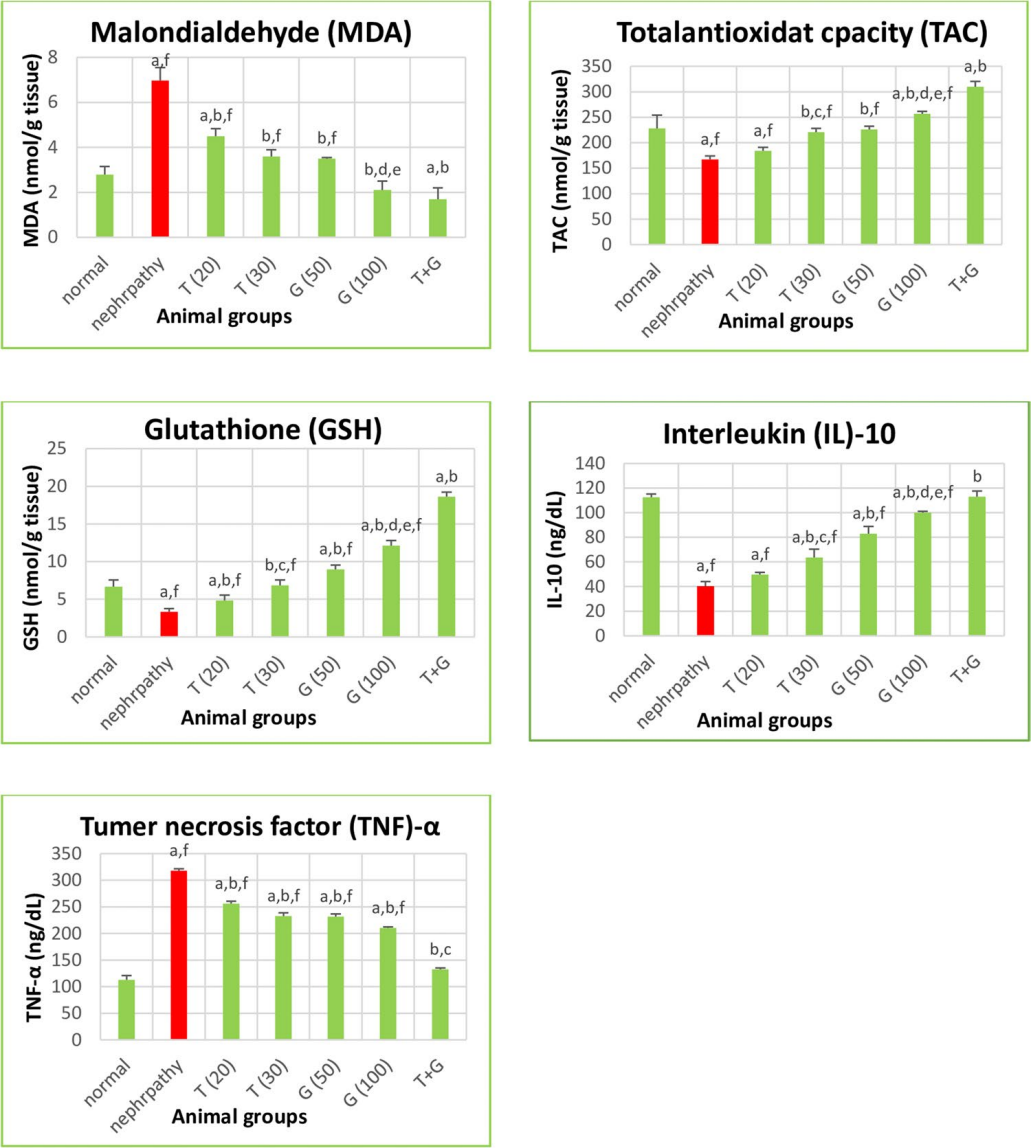
Effect of different treatments on antioxidants, lipid peroxidation, and inflammation in different study groups. Data are expressed as mean ± SE, *n* = 6 rats. Test: ANOVA test followed by Tukey test. *P* < 0.05 is significant. (a) *P* < 0.05 against normal, (b) *P* < 0.05 against nephropathy, (c) *P* < 0.05 against T (20), (d) *P* < 0.05 against G (50), (e) *P* < 0.05 against T (30), (f) *P* < 0.05 against T + G

TAC, GSH, and MDA levels were investigated to measure oxidative stress and antioxidant status as well as TNF-α and IL-10 levels were investigated to measure inflammation. Firstly, TAC and GSH levels were decreased while MDA levels were elevated in the nephropathy group. Notably, significant elevations in TAC and GSH, and reductions in MDA levels were observed across the intervention therapy with thymoquinone and glycine (*P* < 0.05 for all treated groups vs. nephropathy). The dual administration of thymoquinone and glycine led to notable elevations in the levels of antioxidants (GSH and TAC) significantly compared with other treatments (*P* < 0.05). Regarding inflammation, the nephropathy group significantly exhibited elevated TNF-α levels alongside reduced IL-10 levels compared to the control group (*P* < 0.05). Significantly, thymoquinone and/or glycine interventions demonstrated reductions in TNF-α levels and increases in IL-10 levels compared to the nephropathy group (*P* < 0.05), except for T (20), which increased IL-10 insignificantly (*P* > 0.05). Combined treatment (T + G group) displayed more pronounced effects on TNF-α and IL-10 levels. When treated groups were compared with each other, significant (*P* < 0.05) differences were found between T (20) and T (30) in TAC, GSH, and IL-10, between G (50) and G (100) in MDA, TAC, GSH, and IL-10, between T (30) and G (100) in MDA, TAC, GSH, and IL-10, and between T + G and G (100) in TAC, GSH, IL-10, and TNF-α (Fig. 3).

As presented in Table 1, rats with nephropathy showed significant increases in the activities of CK-T and CK-MB (*P* < 0.05 compared to normal rats). When different treatments were applied to the nephropathic rats, significant decreases in these enzyme activities were observed (*P* < 0.05 for all treated groups compared to the nephropathy group). The most effective treatment was the combination of thymoquinone and glycine. Significant differences (*P* < 0.05) were noted between the following treatment groups: T (20) and T (30), G (50) and G (100), T (30) and G (100), as well as between T + G and G (100).

**Table 1:** Effect of different treatments on creatine kinase (CK) enzymes in different study groups

Groups	CK-T (IU/L)	CK-MB (IU/L)
Normal	576 ± 52.2	847 ± 18.03
Nephropathy	2166 ± 329^a,f^	2006 ± 115.8^a,f^
T (20)	1420 ± 39.24^a,b,f^	1637 ± 50.7^a,b,f^
T (30)	1174 ± 28.36^a,b,c,f^	1387 ± 55.9^a,b,c,f^
G (50)	1048 ± 55.56^a,b,f^	1281 ± 27.3^a,b,f^
G (100)	812.7 ± 29.14^a,b,d,e,f^	975 ± 20.6^a,b,d,e,f^
T + G	567.7 ± 27.22^b^	766 ± 110.6^b^

Data are expressed as mean ± SE, *n* = 6 rats. Test: ANOVA test followed by Tukey test. *P* < 0.05 is significant. ^a^*P* < 0.05 against normal, ^b^*P* < 0.05 against nephropathy, ^c^*P* < 0.05 against T (20), ^d^*P* < 0.05 against G (50), ^e^*P* < 0.05 against T (30), ^f^*P* < 0.05 against T + G

**Fig. 4:**
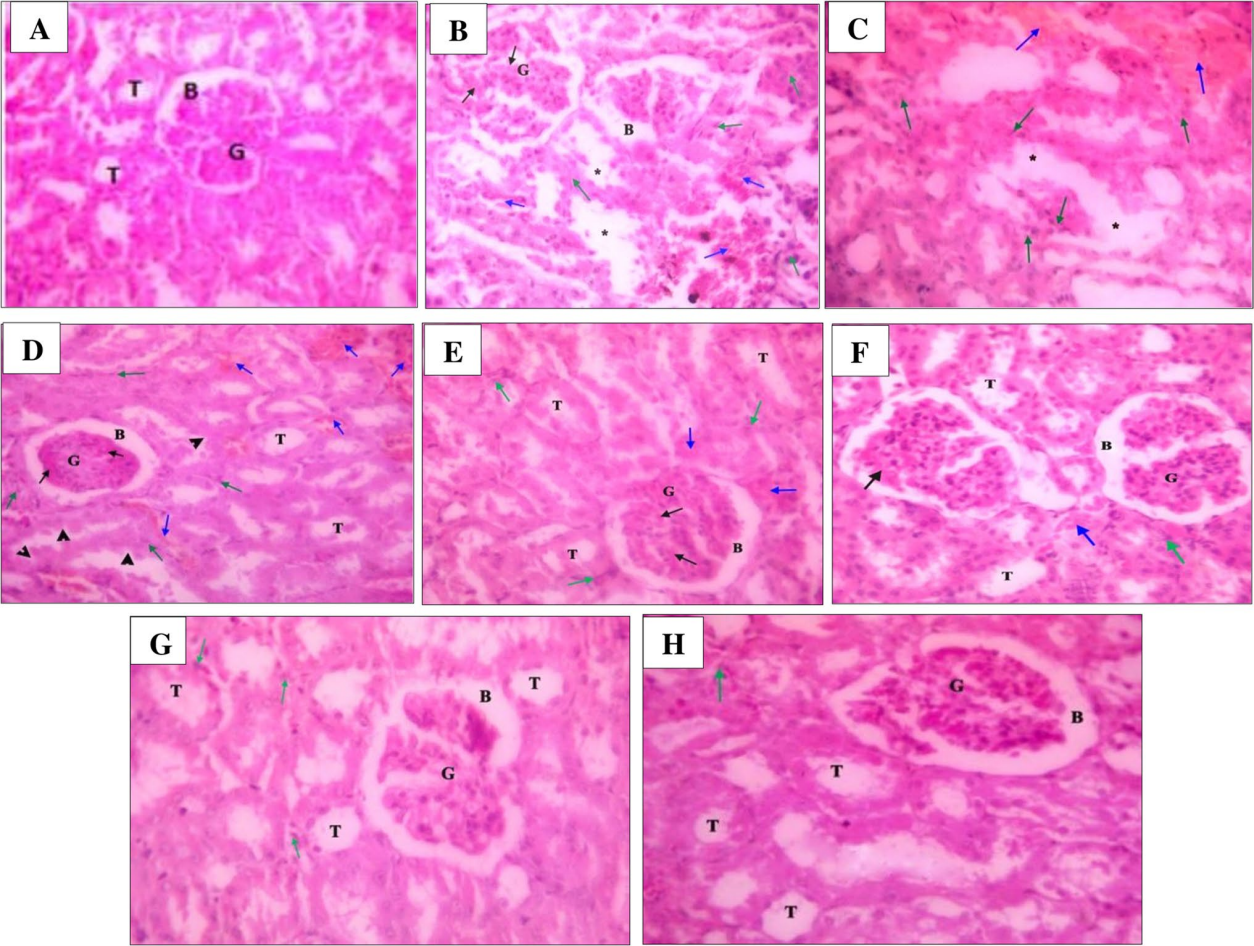
Histological sections of the kidney of different study groups. **A** Kidney section of normal control group showing normal renal architecture represented by normal glomeruli (G), Bowman’s space (B), and renal tubules (T). **B** and **C** STZ-induced nephropathy rats sections showing damaged renal tissue with congestion of the glomerular tuft (black arrow), interstitial hemorrhage (blue arrow), interstitial infiltration (green arrow), and necrosis of the renal architecture (star). **D** T (20) section of rats with nephropathy treated with thymoquinone (20 mg/kg) showing slight restoration of normality of renal tissue with atrophied glomeruli (G), congestion of the glomerular tuft (black arrow), interstitial hemorrhage (blue arrow), interstitial infiltration (green arrow) and degeneration of lining epithelium of renal tubules (head arrow). **E** T (30) section of rats with nephropathy treated with thymoquinone (30 mg/kg) showing moderate restoration of normality of renal tissue with, congestion of the glomerular tuft (black arrow), interstitial hemorrhage (blue arrow) and interstitial infiltration (green arrow). **F** G (50) section of rats with nephropathy treated with glycine (50 mg/kg) showing moderate restoration of normality of renal tissue; still showing congestion of the glomerular tuft (black arrow), interstitial hemorrhage (blue arrow) and interstitial infiltration (green arrow). **G** G (100) section of rats with nephropathy treated with glycine (100 mg/kg) showing restoration of normality of renal tissue except for the moderate interstitial infiltration (green arrow). **H** T + G section of rats with nephropathy treated with a combination of thymoquinone (30 mg/kg) and glycine (100 mg/kg) showing restoration of normality of renal architecture except for slight interstitial infiltration (green arrow), H&E stain

Figure 4 shows the impact of different treatments on kidney tissue. The kidney section of the normal control group showed normal renal architecture with normal glomeruli, Bowman’s space, and renal tubules. STZ-induced nephropathy rats sections showed damaged renal tissue with congestion of the glomerular tuft, interstitial hemorrhage, interstitial infiltration, and necrosis of the renal architecture. Section of rats with nephropathy treated with thymoquinone (20 mg/kg) showed slight restoration of normality of renal tissue with atrophied glomeruli, congestion of the glomerular tuft, interstitial hemorrhage, interstitial infiltration, and degeneration of lining epithelium of renal tubules. Section of rats with nephropathy treated with thymoquinone (30 mg/kg) showed moderate restoration of normality of renal tissue with congestion of the glomerular tuft, interstitial hemorrhage, and interstitial infiltration. Section of rats with nephropathy treated with glycine (50 mg/kg) showed moderate restoration of normality of renal tissue but still showed congestion of the glomerular tuft, interstitial hemorrhage, and interstitial infiltration. Section of rats with nephropathy treated with glycine (100 mg/kg) showed restoration of normality of renal tissue except for the moderate interstitial infiltration. Finally, the section of rats with nephropathy treated with the combination of thymoquinone (30 mg/kg) and glycine (100 mg/kg) showed restoration of normality of renal architecture except for slight interstitial infiltration.

## Discussion

Diabetic nephropathy is manifested by alterations in kidney architecture and function (Jin et al. 2023). The nephropathy caused by STZ is well-documented and primarily results from ROS formation, which triggers inflammation and oxidative stress (Rehman et al. 2019). Under diabetic conditions, diabetes-triggered metabolites, including glucose, inflammatory cytokines, and oxidative insult have been shown as the chief participants in the onset and progression of diabetic nephropathy (Hsu et al. 2016; Miranda-Diaz et al. 2016). Therefore, natural compounds that have anti-inflammatory and antioxidant activities are probable candidates for treating diabetic nephropathy.

In agreement with Pournaghi et al. (2012), the nephropathy group displayed a substantial reduction in body weight, indicative of the weight loss commonly associated with diabetes-related metabolic dysregulation, a phenomenon attributed to the impact of diabetes on the gastrointestinal tract, hindering the efficient absorption of nutrients from ingested food. Other potential causes include dehydration and the degradation of proteins and lipids (Kumar et al. 2018). Notable improvements following administration of thymoquinone, or glycine, or the combination in particular suggest the ability of each to mitigate diabetes-associated weight loss and indicate a synergistic effect between thymoquinone and glycine. Thymoquinone has been shown to improve the diabetic phenotype by lowering glucose and insulin levels while enhancing glucose tolerance, thereby preserving body weight and lipid profile (Zinvand Lorestani et al. 2023). Similarly, El-Hafidi et al. (2018) indicated that glycine supplementation improves insulin sensitivity and glucose tolerance.

STZ-induced nephropathy resulted in a significant increase in kidney weight, which indicates kidney hypertrophy that might occur due to the stimulation of protein synthesis (Habib 2018) or may be caused by inflammation. Our results are consistent with those reported by Lomas-Soria et al. (2011). In contrast, kidney weight remained within the normal range in rat groups that received various treatments, suggesting an effective inhibition of kidney hypertrophy. This maintenance may be attributed to the antioxidative properties, ability to regulate inflammatory responses, impact on electrolyte and fluid balance, and effects on renal function, as indicated below.

The kidney index is an important indicator of renal health, showing a higher value under diabetic conditions, demonstrating kidney hypertrophy (Sun et al. 2024). Reductions in this index caused by treatments with thymoquinone, glycine, or both suggest a potential reversal or attenuation of diabetes-induced renal complications.

Blood analysis of untreated rats injected with STZ revealed signs of uremia; elevated blood levels of urea, creatinine, and uric acid, demonstrating diabetic nephropathy (Aboulqassim and Talib 2023), in which the body depends on proteins instead of carbohydrates as an alternative source for energy resulting in increasing non-protein nitrogenous compounds in blood. Uremia may also occur due to reduced renal excretion of urea and creatinine, resulting from a decrease in glomerular filtration rate attributed to renal dysfunction (Saad et al. 2018). Additionally, there were disturbances in electrolyte levels manifested by hypernatremia, hyperchloremia, hypercalcemia, hypokalemia, and hypophosphatemia. Hypokalemia may result from osmotic diuresis and/or diabetic-induced intestinal malabsorption (Liamis et al. 2014). Hypophosphatemia might occur due to a transcellular shift, osmotic diuresis, and reduced renal phosphate reabsorption (van der Vaart et al. 2021). Hypercalcemia could be attributed to hypophosphatemia, hyperparathyroidism, or dehydration (Liamis et al. 2014). Under diabetic conditions, serum sodium levels fluctuate according to the equilibrium between hyperglycemia, which provokes water to get out of cells and decreases sodium levels, and glucosuria, which promotes osmotic diuresis and raises sodium levels (Alqubaty et al. 2022). It was also reported by Engwa et al. (2018) that increased serum chloride may be due to hypertonicity or ketoacidosis, as noted by Majid et al. (2018). Glycine and thymoquinone therapies caused improvements that may be attributed to improvements in renal function, normalization of metabolic processes, regulation of fluid balance, anti-inflammatory effects, and modulation of mineral channels, aligning with Graczyk et al. (2024).

High glucose encourages glucose oxidation and more production of free radicals in mitochondria, leading to oxidative stress, which damages DNA, increasing apoptosis, which is regarded as a great initiator of kidney dysfunction accompanying diabetes (Giacco and Brownlee 2010; Ghasemi et al. 2019). Thymoquinone possesses potent antioxidant and anti-inflammatory properties, which can attenuate oxidative stress and inflammation-induced renal injury (Li et al. 2022). Similarly, glycine, an amino acid with cytoprotective properties, may help mitigate oxidative stress (Shosha et al. 2023) and modulate the inflammatory responses in the kidneys (Shafiekhani et al. 2019). Our study confirmed these findings, showing that various treatment protocols revealed improvements in MDA, GSH, and total antioxidant levels. A recent study of Hosseini and his colleagues (2024) reported findings similar to ours regarding rhabdomyolysis-induced acute kidney injury, where thymoquinone treatment decreased creatinine levels, mitigated histopathological changes, reduced MDA levels, and increased GSH amounts.

TNF-α promotes inflammation and immune responses, while IL-10 counteracts excessive inflammation, promoting immune regulation and tissue repair. This balance between TNF-α and IL-10 is crucial for proper immune system functioning and maintaining immune homeostasis. The dysregulated TNF-α and IL-10 signaling is implicated in various inflammatory and autoimmune diseases. In rats with diabetic nephropathy, we observed increased TNF-α, a pro-inflammatory cytokine, and decreased IL-10. Interventions with thymoquinone, glycine, or their combination resulted in reduced TNF-α and increased IL-10, indicating an immunomodulatory effect. This supports the studies of Zhang et al. (2024), who indicated that lowering inflammation may contribute to thymoquinone’s ability to shield the kidneys, and Shosha et al. (2023), who observed glycine’s capacity to lower TNF-α. However, this contrasts with the conclusions of Hosseini et al. (2024), who found that thymoquinone had no significant impact on TNF-α.

Diabetes disrupts essential metabolic pathways related to energy production and muscle function. The impairment of kidney function due to diabetic nephropathy leads to metabolic imbalances that markedly affect muscle tissues. CK is a key biomarker for muscle tissue injury (Sofiullah et al. 2024). It leaks from the cytosol and increases in circulation as a result of muscle damage. In this experiment, diabetes-induced nephropathy was associated with high CK-T, indicating diabetes-associated muscle injury/or stress, and CK-MB, which suggests heart problems reflecting the detrimental effects of diabetes on cardiac function. This may result from various factors, including oxidative stress, inflammation, and diabetes-dependent metabolic abnormalities. Intervention with thymoquinone and/or glycine demonstrated reductions in CK-T and CK-MB indicating a potential cytoprotective effect, especially on the myocardial cells. Thymoquinone or glycine probably mitigates myocardial damage by reducing oxidative stress and inflammation. Our findings align with previous studies, including those by de Meijer et al. (2003) and by Safari et al. (2016), which reported elevated CK levels in patients with acute renal failure, and established a correlation between elevated CK levels and the onset of acute kidney injury, respectively. In addition, the research conducted by Mohamed and Akasha (2019) as well as the research by Jevrić-Čaušević et al. (2006) and by Awadalla et al. (2010) that reported increased total CK activity in diabetic patients.

**Fig. 5:**
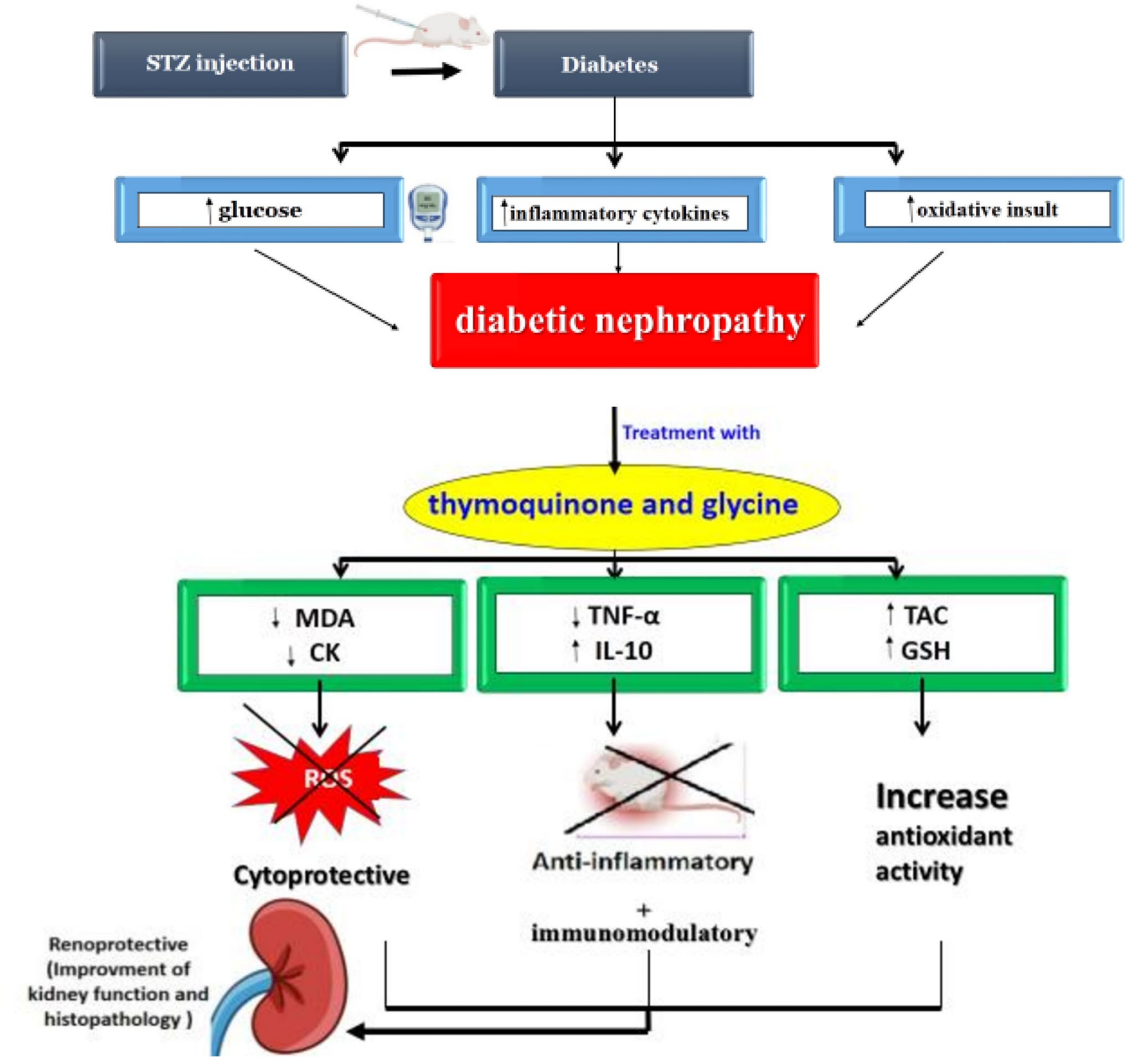
Proposed mechanism for the synergistic management of streptozotocin-induced nephropathy using combined thymoquinone and glycine treatment

Further, the biochemical effects of the different doses of thymoquinone and glycine therapies were compared in this study. Our findings indicate the dose-dependent effects of thymoquinone and glycine and demonstrate that glycine is more effective than thymoquinone with the superiority of their combination. In our experiment, several mechanisms are suggested to be responsible for the observed improvements of the treated nephropathic rats using thymoquinone and glycine. These mechanisms include ROS reduction, antioxidants increase, and cytoprotective, immunomodulatory, and anti-inflammatory effects. Collectively, these factors contribute to enhancements in renal architecture and function (Fig. 5).

Finally, regarding kidney architecture, STZ-induced diabetes is known to induce renal damage primarily through oxidative stress, inflammation, and altered glucose metabolism, leading to impaired renal function (Wang and Zhang 2024). Herein, STZ-induced nephropathy resulted in severe kidney damage characterized by congestion, hemorrhage, and interstitial infiltration. These observed histopathological changes, along with the biochemical results of other diagnostic tests for diabetic nephropathy, underscored the progression of diabetic nephropathy as a prominent complication of diabetes.

Thymoquinone and glycine therapy may exert reno-protective effects by targeting the underlying mechanisms. Similar protective effects via oxidative and histopathological modulation have been reported in toxin-induced models such as cisplatin-induced nephrotoxicity (Bazmandegan et al. 2021). Treatments of nephropathy rat groups with thymoquinone, glycine, or their combination demonstrated varying degrees of restoration of kidney healthy structure in the following order: T + G > G (100) > G (50) > T (30) > T (20). These findings support the dose-dependent effect of thymoquinone or glycine and prove that glycine is more effective than thymoquinone and that combination therapy is the best. Accordingly, we attribute the synergistic effect observed with combined thymoquinone and glycine therapy to their complementary mechanisms of action. This dynamic demonstrates promising outcomes in safeguarding renal structure and function and showcases remarkable antioxidant properties, crucial for combating oxidative stress—a hallmark of diabetic nephropathy.

The limitations of this study include a fairly small sample size, which restricts our ability to generalize conclusions. We utilized only one animal model of diabetic-induced nephropathy. Unfortunately, we were unable to extend the duration of the drug treatment. To enhance reliability, future research is crucial to validate our findings. Further studies should involve larger sample sizes and varied models of diabetic nephropathy using different rodent species. Additionally, the mechanisms underlying the synergistic effects of thymoquinone and glycine require further investigation in more depth. Future studies should also evaluate the safety and efficacy of these treatments for managing diabetic nephropathy over longer periods for more validation of the results.

## Conclusion

Our findings demonstrated signs of STZ-induced diabetic nephropathy in rats: weight loss, kidney hypertrophy, elevations of serum creatinine, urea, and uric acid levels, disturbances in mineral levels alongside oxidative stress, and inflammatory deterioration. Glycine or thymoquinone alone or combined ameliorated these pathological alterations. The combination of glycine and thymoquinone exhibited the most effective therapeutic effects. The curative effects of glycine or thymoquinone alone or combined in treated diabetic nephropathy rats are suggested to be linked to their antioxidative, immunomodulatory, cytoprotective, and anti-inflammatory properties. Histopathological studies supported our biochemical findings. These results highlight the synergistic benefits of combining thymoquinone and glycine to alleviate diabetic nephropathy.

## Data Availability

All source data for this work (or generated in this study) are available upon reasonable request.
